# Antitumor activity of membranes associated with *Acmella oleracea* extract

**DOI:** 10.1590/1414-431X2024e14129

**Published:** 2024-11-04

**Authors:** C.A. Priante-Silva, B.H. Godoi, R.F. Menegon, N.S. da Silva, C. Pacheco-Soares

**Affiliations:** 1 Instituto de Pesquisa e Desenvolvimento, Laboratório de Dinâmica de Compartimentos Celulares, Universidade do Vale do Paraíba, São José dos Campos, SP, Brasil; 2 Instituto de Pesquisa e Desenvolvimento, Laboratório de Fotobiologia Aplicada è Saúde, Universidade do Vale do Paraíba, São José dos Campos, SP, Brasil; 3 Laboratório de Insumos Naturais e Sintéticos, Universidade Federal de São Paulo, Diadema, SP, Brasil; 4 Universidade Estadual de São Paulo Júlio de Mesquita Filho, São José dos Campos, SP, Brasil

**Keywords:** Biomaterial, Chitosan, HEp-2 culture, Cancer, Extract

## Abstract

Epithelial cancers, such as epidermoid cancer and some adenocarcinomas, affect surface areas that are generally more accessible to various treatments. However, this group of tumor cells has an aggressive behavior, leading to a high annual mortality rate. The development of a biomaterial that is non-invasive, can kill tumor cells, and prevent opportunistic infections is the basis for the treatment for this type of cancer. Therefore, the objective of this study was to develop a biomaterial from chitosan and *A. oleracea* extracts that exhibits cytotoxic action against the HEp-2 tumor cell line. Dried crude 90% ethanol extracts were obtained through ultrasound-assisted maceration, followed by liquid-liquid extraction to yield the butanol fraction. From these extracts, chitosan membranes were developed and evaluated for their antitumor activity against HEp-2 using viability tests with crystal violet and MTT (3-(4,5-dimethylthiazol-2-yl)-2,5-diphenyltetrazolium bromide) assay, in addition to a wound healing test. The cytotoxic assays indicated a significant reduction in cell density and mitochondrial activity, especially at the concentration of 1000 µg/mL of crude extract. The butanol fraction had minimal effects on mitochondrial activity. The wound healing test demonstrated that the biomaterial and extract prevented closure of the wound created in the cell monolayer within 48 h of incubation and caused changes in cell morphology. In view of this, we concluded that a chitosan membrane associated with a 90% ethanol extract of *Acmella oleracea* exhibited cytotoxic activity is a potential alternative treatment for superficial cancers.

## Introduction

Surface cancers, such as epidermoid cancer or adenocarcinoma, require multiple procedures and rapid healing. These cancers arise from abnormalities in the epithelial cells of the skin or mucosa and are very common in oral and non-melanoma skin cancers (1).

Radiotherapy combined with surgery is the most effective treatment for cancer. In the initial I and II stages, surgery alone is indicated as treatment, being effective without significant aesthetic alterations. However, in the more advanced stages III and IV, radiotherapy and invasive surgery as well as additional corrective procedures are required to address the wounds left after the treatment.

Therefore, alternative treatments that use biomaterials in combination with medicinal plants are of great interest. Membranes composed of bioactive extracts act as a barrier and help to eradicate tumor cells and microorganisms, restore tissue, and prevent potential infections by opportunistic pathogens (2).

Chitosan membranes are among the biomaterials commonly used in association with bioactive substances. These compounds have an efficient surface for cell adhesion, are inert at low concentrations, and show no positive or negative effects, such as allergic processes. These characteristics allow these membranes to be combined with various active substances that stimulate activities such as cell proliferation and antimicrobial and anesthetic activities. They are also used in hydrogels, cements, and membranes for various medical applications, including healing, osteoinduction, combating pathogenic microorganisms in implants, and as scaffolds for other medications (3-5).

A promising plant species native to Brazil that combines healing, antimicrobial, and antitumor characteristics is *Acmella oleracea*, commonly known as jambu. Jambu is easy to cultivate and adapts well to various climates. In addition, *A. oleracea* is a suitable component of biomaterials for tissue recovery, making it a non-invasive and cost-effective candidate for antitumor treatment (6-8).

Da Silva (9) conducted a study involving the biomonitoring fractionation of jambu leaves, starting with a 90% crude ethanolic extract, followed by subsequent liquid-liquid extractions in ascending order of the extracting solvent's polarity, resulting in hexane, chloroform, ethyl acetate, n-butanol, and water fractions. The cytotoxic properties were tested individually, with the most intense cytotoxic activities being obtained from the ethyl acetate and n-butanol fractions. These fractions were further separated using molecular exclusion column chromatography (Sephadex-LH20, Sigma-Aldrich - MilliporeSigma, USA) and analyzed using high-performance liquid chromatography with photodiode-array detection (HPLC-DAD). In these analyses, the compound spilanthol could not be identified, but it was observed that while the ethyl acetate fraction was almost entirely composed of phenolic acid derivatives, the n-butanol fraction contained coumarinic acid, ferulic acid, flavonoids, and derivatives of caffeic and phenolic acids, to which the cytotoxic properties observed for the ethyl acetate and especially the n-butanol fraction of *Acmella oleracea* were attributed.

Furthermore, our group studied the effect of *A. oleracea* on actin polymerization and filament formation in HEp-2 and L929 cells (mouse fibroblast subcutaneous connective tissue) using a 90% hydroethanolic extract (10). The influence on cytoskeleton organization was clearly affected by *A. oleracea* extract in a dose-dependent manner, showing depolymerization of actin filaments, loss of morphology, and compromised cell adhesion in HEp-2 cell lines. However, a lesser effect was observed on the mammalian L929 cell line at the same concentration (500 µg/mL), indicating a possible selectivity between mammalian and tumor cell lineages.

The healing activity of the ethyl acetate and n-butanol fractions was also studied by da Silva (11), wherein it was observed that the healing activity of both fractions could be confirmed starting at a concentration of 250 µg/mL for L929 cells. However, the wound in the HEp-2 tumor cell culture was not completely closed even after 48 h of incubation, demonstrating significant potential in promoting the recovery of healthy tissue but not tumor tissue.

In light of this, we aimed to explore the potential use of the n-butanol fraction of *A. oleracea* leaf extract for the treatment of surface tumors by observing the cytotoxic effects of chitosan membranes loaded with both the crude extract and the n-butanol fraction, as well as the healing activity of these biomaterials on mammalian L929 and HEp-2 tumor cells.

## Material and Methods

**Figure 1:**
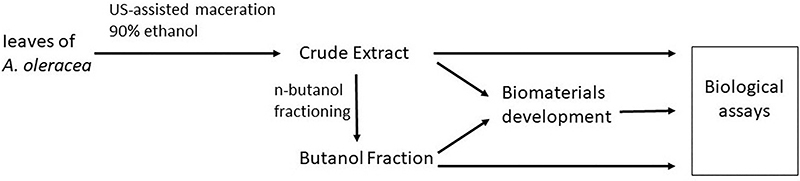
Schematic representation of the extraction of *A. oleracea* extracts and the development of biomaterials for biological assay. US: ultrasound.

*In vitro* studies of cytotoxicity and mitochondrial viability in HEp-2 tumor lineage cells were conducted using biomaterials developed with crude and butanolic extracts of *A. oleracea* leaves. Figure 1 shows a schematic representation of the entire sample preparation process and the biomaterial and biological assays, which are detailed in the subsequent sections.

### Preparation of the plant extract

The sowing of *Acmella oleracea* (L.) RK Jansen took place in a greenhouse equipped with an irrigation system and shade net to ensure the necessary supply of water and adequate light for plant development. These greenhouses are located at the Nature Studies Center (CEN - Centro de Estudos da Natureza) of the University of Vale do Paraíba (UNIVAP) in Brazil, with temperatures ranging between 17 and 29.5°C in the first semester of 2017. The plant was identified by Prof. Dr. Renata Jimenez de Almeida Scabbia from the University of Mogi das Cruzes (UMC) and deposited in the Herbarium Mogiense (UMC) with the record number HUMC 6847.

Leaves of the plants were dried in an oven at 50°C, pulverized, and extracted using ultrasound-assisted maceration (USM) with 90% ethanol at a ratio of 1:10 (mass of plant/volume of extractor liquid) for 60 min. The leaves were macerated for seven days and then evaporated until dry under reduced pressure.

The crude extract was resuspended in distilled water and fractionated using n-butanol as a solvent. After drying under reduced pressure, the butanol fraction was stored at -20°C. The dried extracts (butanol and crude) were diluted in Dulbecco's Modified Eagle's medium (DMEM) to an appropriate concentration and supplemented with 10% fetal bovine serum (FBS) and 1% antibiotics. For cytotoxicity assays in HEp-2 cells, extract concentrations of 250, 500, and 1000 µg/mL were used, with a control group receiving no extract treatment.

### Development of the biomaterial

The biomaterial, in the form of a membrane, was produced according to Oliveira (12) using 2% chitosan and 1% (m/v) acetic acid with magnetic stirring for 30 min at room temperature. It was then combined with gelatin 4% (m/v), which had been previously dissolved in distilled water at 40°C for 30 min under magnetic stirring. The two solutions were mixed in a 1:1 (v/v) ratio with magnetic stirring at room temperature for 1 h, followed by the addition of glycerin at a concentration of 0.5 % (v/v) and continued magnetic stirring for 1 h. Additionally, the butanol fraction and crude extract were incorporated at a final concentration of 1000 µg/mL, prepared as described previously, resulting in a final membrane containing the plant samples. To evaporate the solvent, 30 mL of the final solution was transferred to a mold-free container and dried at 50°C for 48 h. For biological assays, the samples were supplemented with 1 mL DMEM culture and stirred at 37°C for seven days. The solution was then stored at room temperature.

### Sample groups

From the samples containing the plant extract, test groups were established for the crude extract and butanol fraction at concentrations of 250, 500, and 1000 µg/mL, along with a control group (without extract) for the cytotoxicity assay and at 1000 µg/mL for the healing test. Furthermore, the biomaterial was diluted in DMEM at a 1:1 ratio (membrane:mL) to establish sample groups of the chitosan membrane (without added extract), chitosan membrane with crude extract (1000 µg/mL), and chitosan membrane with butanol fraction (1000 µg/mL). These preparations were then used to treat cells.

### Crystal violet assay

The HEp-2 cell line (human carcinoma, ATCC CCL-23, Institute Adolfo Lutz-Cell Culture Section, Brazil) was cultured in 48-well plates (1×10^5^ cells) using mL-1 (TPP, Switzerland) with DMEM (Dulbecco's Eagle's Modified Medium, Gibco L, USA) supplemented with 10% fetal bovine serum (FBS, Gibco BRL) and 1% antibiotic (Gibco BRL) and incubated at 37°C at 5% CO_2_ for 24 and 48 h at the concentrations described previously. In each well, 5% crystal violet solution (Sigma^®^, Brazil) was added and incubated for over 3 min. The plates were then washed in running water for 2 min and subsequently 1% sodium dodecyl sulfate (SDS) solution was added to the wells and incubated for 1 h at room temperature. Absorbance was measured at 570 nm using a spectrophotometer (ELISA SpectraCount, Packard, USA). The test was performed in triplicate. The values obtained were transformed into percentages using the following formula: (Control absorption - 0.045) / (Sample absorption - 0.045) × 100, where 0.045 represents the absorbance reading of a plate well without a sample or culture medium (13).

### MTT assay

For the MTT bromide [3-(4,5-dimethylthiazol-2-yl)-2,5-diphenyltetrazolium bromide] (Sigma^®^) assay, cells were transferred at a density of 1×10^5^ per well in 48-well plates and then treated with the dilutions of the extract and diluted membranes and incubated for 24 h. After incubation, 200 µL of MTT (5 mg/mL) was added and incubated for 60 min at 37°C; then, 200 µL of DMSO (dimethylsulfoxide, Sigma^®^) was added to the wells and stirred for 60 min. Absorbance was measured at 570 nm using a spectrophotometer (ELISA Spectroradiometer, Packard). The test was performed in triplicate. The values obtained were transformed into percentages using the following formula: (Control absorption - 0.045) / (Sample absorption - 0.045) × 100, where 0.045 represents the absorbance reading of a plate well without a sample or culture medium (14).

### Wound healing test

Cells were transferred to 24-well plates at a density of 5×10^5^ cells/well. After incubation for 24 h in an oven at 37°C and 5% CO_2_ in DMEM, a wound was created on the cell monolayer. Plant extracts and diluted membranes were added to these wells at a concentration of 1000 µg/mL. The wells were photographed at the incubation times of 0 (control), 24, and 48 h using an optic microscope (15).

### Statistical analysis

For statistical analysis of the feasibility tests, the data were normalized and converted into a population percentage of cells.

A two-way analysis of variance (ANOVA) test followed by the Tukey test with a significance of P<0.05 were used to analyze the variance of the groups as a function of the evaluation times of 24 and 48 h.

GraphPad Prism 6^®^ software (USA) was used to perform the statistical analysis and plot the graphs. The data are reported as medians and interquartile range, where a statistical difference between the control and test groups was illustrated using a standard scoring system.

## Results

**Figure 2:**
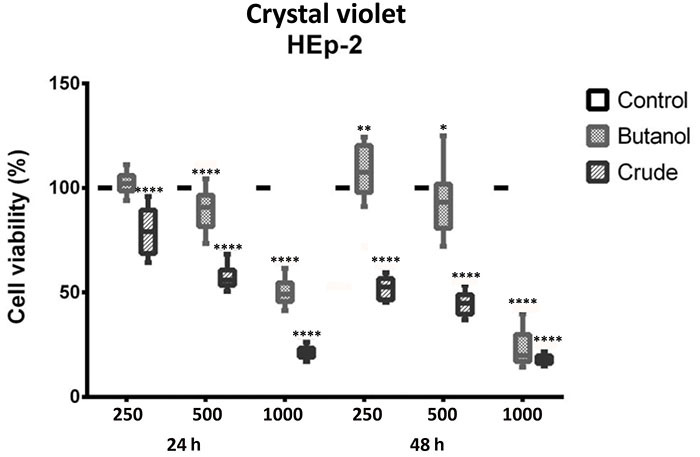
Cell viability by crystal violet assay at 24 and 48 h of HEp-2 cells incubated with the crude extract and butanol fraction of *A. oleracea*. Crude extract in both evaluation periods caused a decrease in cell density, observed at the three study concentrations (250, 500, and 1000 µg/mL). Data are reported as median and interquartile range. *P<0.05, **P<0.01, ****P≤0.0001 compared to control; ANOVA.

**Figure 3:**
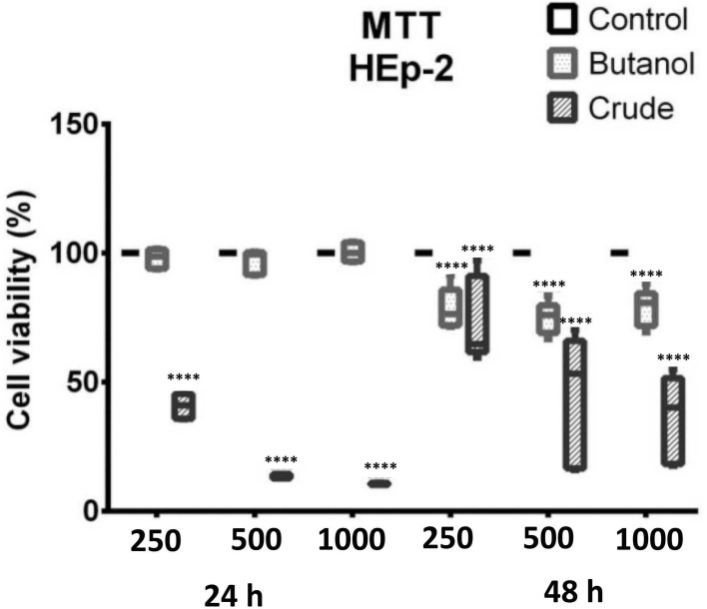
3-(4,5-dimethylthiazol-2-yl)-2,5-diphenyltetrazolium bromide (MTT) assay demonstrating the effect of *A. oleracea* extract on the growth of HEp-2 cells after 24 and 48 h of incubation with the crude extract and butanol fraction. The data are reported as median and interquartile range. At all concentrations (250, 500, and 1000 µg/mL) and both time points evaluated, the butanol group demonstrated a decrease in mitochondrial activity after 48 h with a statistical significance Data are reported as median and interquartile range. ****P≤0.0001 compared to control; two-way ANOVA (Bonferroni post-test).

The crystal violet assay demonstrated that both extracts behaved in a dose-dependent manner, but with varying effects depending on the exposure time. While the crude extract caused a significant decrease (P≤0.05) in the population density of HEp-2 cells at all tested concentrations, the n-butanol fraction did not interfere with the population density of these cells at the lowest tested concentration (250 µg/mL). However, the cytotoxic effect of the n-butanol fraction emerged at the concentration of 500 µg/mL in a dose-dependent manner (Figure 2). After 48 h of incubation, the decrease in cellular population density was equal for both extracts at the highest tested concentration (1000 µg/mL) (Figure 3). Yet, while the cytotoxic action remained pronounced for the crude extract at the other concentrations, this effect appeared slightly reversed with the n-butanol fraction, even suggesting a population growth at the concentration of 250 µg/mL.

When analyzing the mitochondrial activity using the MTT assay, the differences between the two extracts became quite pronounced, demonstrating a marked effect of the crude extract at all tested concentrations over 24 and 48 h of incubation. This action on mitochondrial activity did not occur with the butanolic fraction, which showed no observable action over 24 h of incubation, and only a modest reduction in mitochondrial activity (P≤0.0001) after 48 h. Such divergence between the activities may be attributed in part to the distinct chemical composition caused by fractionation with butanol, which concentrates highly polar compounds, such as high sugar derivatives of coumarinic, flavonoid, and phenolic compounds, in contrast to the crude extract, which assembles both low- and high-polarity molecules.

**Figure 4:**
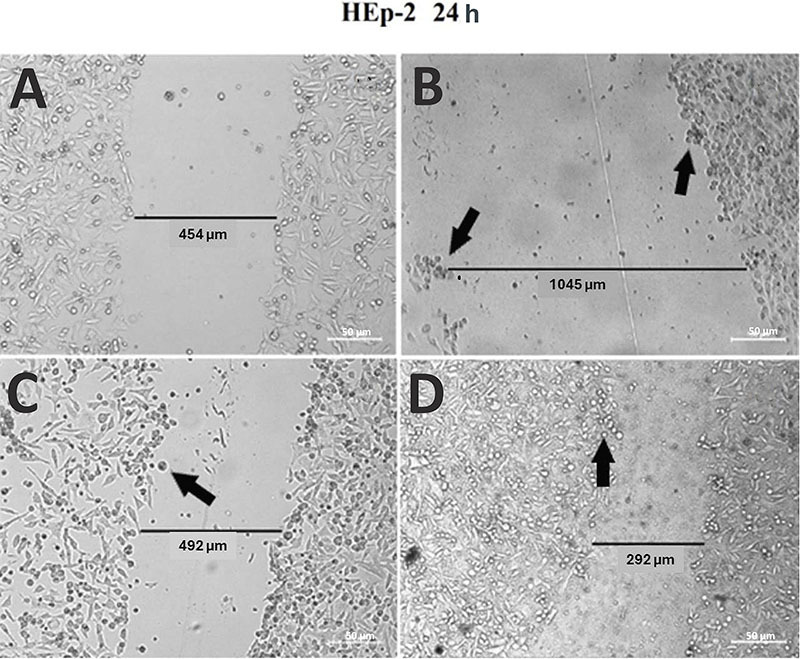
Wound closure test after treatment with butanol and crude extract fractions of *A. oleracea* within a 24-h incubation period. **A**, Control (without addition of extract); **B**, original injury (without addition of extract at 0 h for comparison); **C**, cells and butanol extract; and **D**, cells and crude extract. The arrows point to cells with a change in their morphology resulting in the loss of their ideal viable cell shape. Scale bar 50 µm.

**Figure 5:**
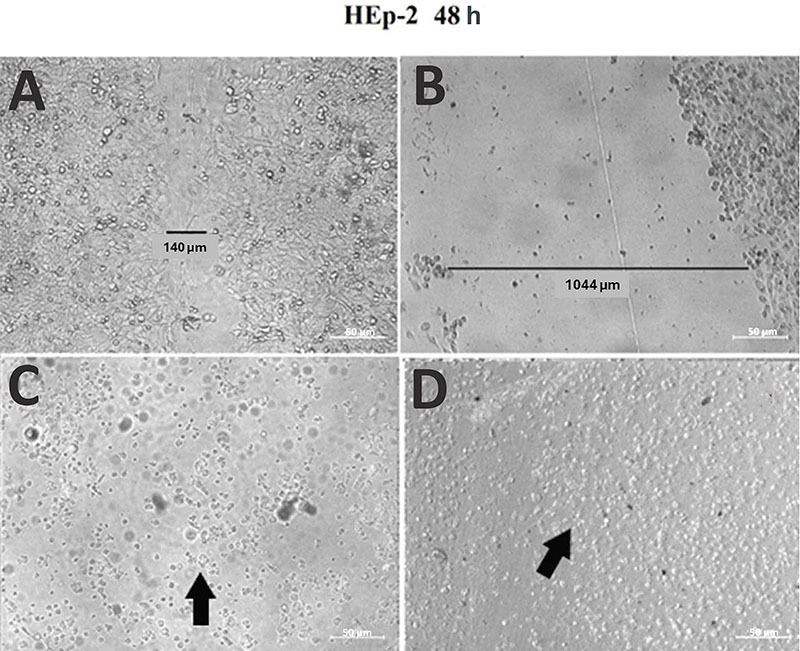
Wound healing test after treatment with butanol fraction and crude extract of *A. oleracea* within 48 h of incubation. **A**, Control (without addition of extract); **B**, original injury (without addition of extract at 0 h for comparison); **C**, cells and butanol extract; and **D**, cells and crude extract. The arrows point to cells with a change in their morphology resulting in the loss of their ideal viable cell shape. Scale bar 50 µm.

The last assay conducted using extracts from *A. oleracea* was the wound healing test, which involved analyzing the ability of a cellular monolayer culture to recover and heal after an injury was created on the cellular culture under experimental conditions. We aimed to evaluate the effect of the extract under analysis on the growth rate of the cellular culture and analyze the morphology of the cells after the incubation period. None of the samples showed wound closure or “healing” of the induced injury even after 48 h of incubation. After 24 h of incubation, an increase in the number of cells was sufficient to delineate the injury compared to that in the control group (Figure 4). However, after 48 h, damage to the cells was observed with a change in their morphology and a decrease in cell density in all samples, which was more pronounced in the crude extracts (Figure 5).

These results are consistent with the expected action of these *A. oleracea* extracts in inhibiting proper actin polymerization and filament formation, which are essential for the formation of the cytoskeleton and thus the preservation of morphology (10).

**Figure 6:**
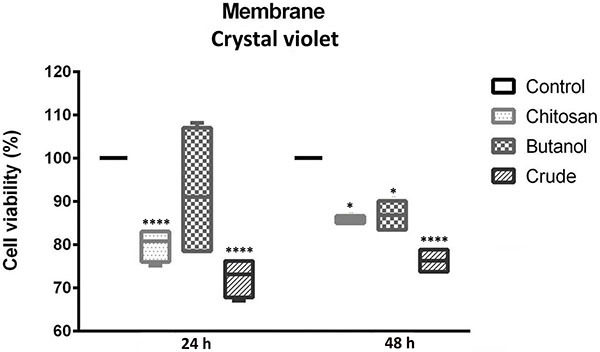
Crystal violet assay within 24 and 48 h of incubation of HEp-2 cells with chitosan membrane and chitosan with butanol and crude extracts of *A. oleracea*. Data are reported as median and interquartile range. *P<0.05, ****P≤0.0001 compared to control; ANOVA.

**Figure 7:**
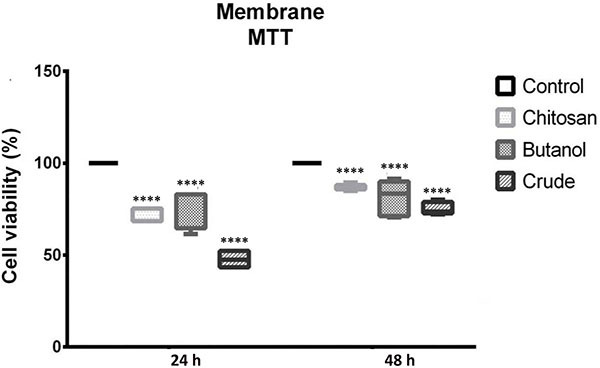
3-(4,5-dimethylthiazol-2-yl)-2,5-diphenyltetrazolium bromide (MTT) assay demonstrating the effect of the extract of *A. oleracea* on the growth of HEp-2 cells. MTT assay within 24 and 48 h of incubation of HEp-2 cells with chitosan membrane and chitosan with butanol and crude extract.. Data are reported as median and interquartile range. ****P≤0.0001 compared to control; ANOVA.

Similar results were obtained from assays conducted using either the pure chitosan membrane or the *A. oleracea* extracts at a concentration of 1000 µg/mL, prepared as described in the Material and Methods section. As shown in Figure 6, the pronounced cytotoxic action of the membrane containing the crude extract was maintained in the crystal violet assay. However, the decrease in population density after incubation with the biomembrane containing the butanol fraction was comparable to that observed with the control chitosan membrane. Similar observations were made in the cell viability study (Figure 7), where the activity of chitosan was equivalent to that of the biomembrane containing the butanol extract.

**Figure 8:**
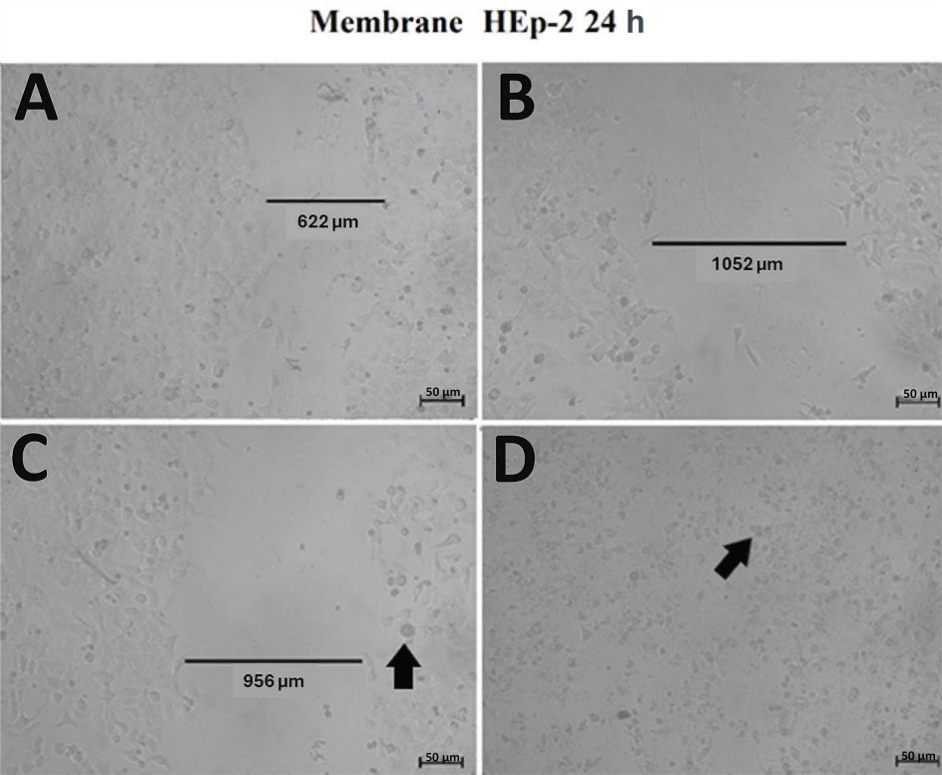
Wound healing test assessing an increase or decrease in the number of cells induced by the membrane-associated chitosan and chitosan crude extract and butanol fraction of *A. oleracea* after an incubation period of 24 h. **A**, Control (without addition of extract); **B**, cells and chitosan membrane; **C**, cells and butanol membrane; and **D**, cells and crude extract membrane. The arrows point to cells with a change in their morphology, resulting in the loss of their ideal viable cell shape. Scale bar 50 µm.

**Figure 9:**
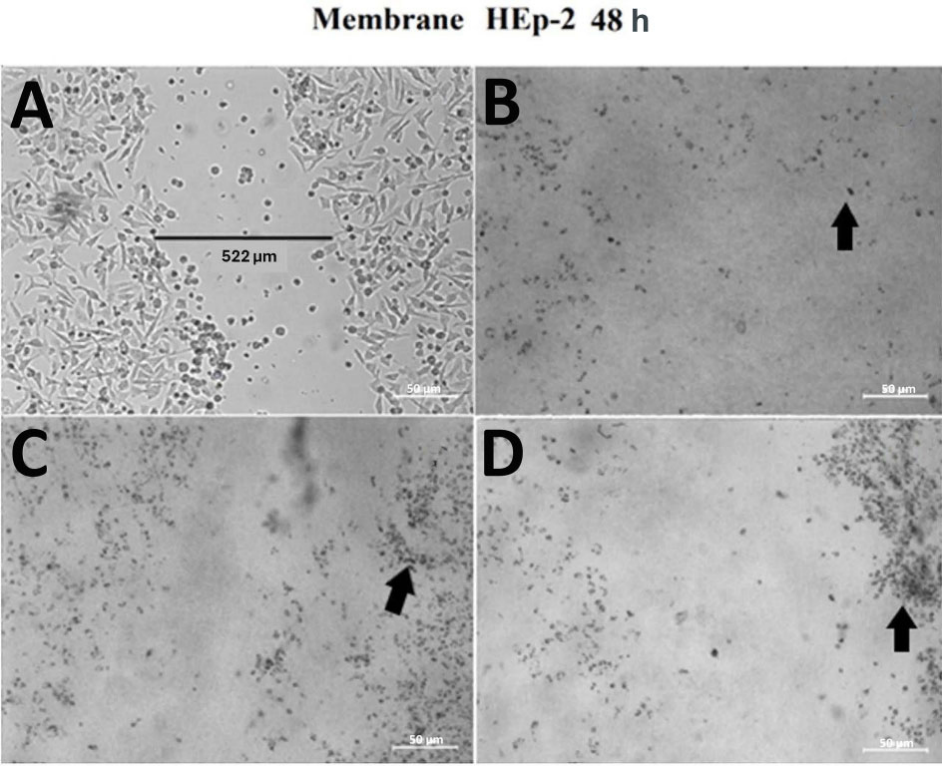
Wound healing test assessing the increase or decrease in the number of cells induced by the membrane-associated to chitosan and chitosan crude extract and butanol at 48 h of incubation. **A**, control (without addition of extract); **B**, cells and chitosan membrane; **C**, cells and butanol membrane; and **D**, cells and crude extract membrane. The arrows point to cells with a change in their morphology, resulting in the loss of their ideal viable cell shape. Scale bar 50 µm.

The cytotoxic activity of chitosan differed from that of the butanol fraction only in the wound healing assay, where, despite none of the membranes allowing complete closure of the injury inflicted on the HEp-2 monolayer, it was possible to observe a change in cellular morphology and likely cell death when the cells were incubated in contact with the diluted membrane associated with the butanol fraction for 48 h and with the crude extract for 24 h (Figure 8) and 48 h (Figure 9) compared to the pure chitosan membrane.

## Discussion

*Acmella oleracea*, indigenous to Brazil, is utilized globally in the cosmetic, pharmaceutical, herbal, and culinary industries. Its biological activity is dose-dependent, exhibiting both toxic and therapeutic effects at varying concentrations (7,16,17). These dual responses are influenced not only by the concentration but also by the extraction methodology, choice of solvent, and specific plant part used (i.e., leaves, roots, or inflorescences). These factors significantly affect the yield of bioactive compounds including alkaloids, flavonoids, coumarins, and phenolic constituents, which are integral to their anti-inflammatory, antioxidant, and antimicrobial properties (6,8,18).

Numerous studies have documented the cytotoxic activity of extracts of *A. oleracea*. These activities are commonly linked to the presence of phenolic compounds, flavonoids, and spilanthol ((2E,6Z,8E)-N-isobutyl-2,6,8-decatrienamide), a distinctive alkylamide of *A. oleracea* sourced from the plant's inflorescences and leaves (8,16,19). Cytotoxic effects are often dose-dependently correlated with flavonoid concentrations (20). Both flavonoids and other phenolic compounds have been posited as potential anticancer agents, primarily due to their antioxidant activity, which reduces free radical formation and neutralizes oxidizing agents (21,22) or induces apoptosis in leukemic cells through the stimulation of cytochrome c release (23,24).

In the current study, we explored the cytotoxic activity of Jambu leaf extracts, as well as the potential to incorporate this extract into a chitosan biomembrane, enabling its therapeutic use as an alternative and non-invasive treatment for surface cancers. We compared the cytotoxic properties of 90% hydroethanolic extract (crude extract) against the fraction containing more hydrophilic compounds obtained through liquid-liquid fractionation with n-butanol.

Naturally, given the high extraction power of the 90% ethanol solution, the resulting crude extract contained a range of phytochemical compounds representing a broad solubility spectrum, from low-polarity substances with few or no sugar molecules to higher-polarity compounds bound to larger sugar fractions, as well as proteins and free sugars (25,26). These more polar hydrophilic compounds were primarily concentrated in the n-butanol fraction.

The cytotoxicity of these fractions was evaluated using two distinct colorimetric methodologies that allowed the observation of events of different nature. Results of both tests are reported as the percentage of cells deemed viable after treatment compared with a control group. Given these differences, the same cell line subjected to the same treatment may yield cell viability inhibition percentages that are not necessarily reproduced in either assay and do not present any direct proportional relationship. However, since both assays reflect the action of the extract on the same cell line over the same period, we recognized the need to observe cytotoxic potential directly through microscopic visualization of growth and regeneration of the cell monolayer and by observing morphological alterations induced by the presence of the extracts.

When observing cell viability using the crystal violet assay, which is based on the interaction between the chromogenic reagent and the genetic material of viable cells undergoing division and adhering to the surface of the culture plate, we clearly observed that the crude extract exhibited greater potency than did the butanol fraction. However, both compounds demonstrated dose-dependent effects on HEp-2 cells, reflecting their expected cytotoxic actions. However, when cell viability was assessed using the MTT assay, the results were quite different.

The MTT assay can be considered an indirect indicator of mitochondrial activity, as it relies on the ability of functioning mitochondria to reduce the tetrazolium salt MTT (3-(4,5-dimethylthiazol-2-yl)-2,5-diphenyltetrazolium bromide) to formazan crystals via NADPH-dependent oxidoreductases. This also reflects the viability of host cells containing these mitochondria, as dead cells or those with compromised mitochondrial function are incapable of reducing MTT to formazan crystals. From this perspective, it is noteworthy that the butanolic fraction, at all tested concentrations, showed no effect on cell viability after 24 h of incubation, with only a slight inhibition similarly observed across the three doses after 48 h, which may reflect actual cell death but through a mechanism that does not involve damage to the mitochondria. In contrast, the crude extract exhibited a strong dose-dependent effect on mitochondrial activity, especially during the first 24 h of incubation. After this period, the results began to show great variance, suggesting a recovery of mitochondrial activity.

Despite the apparent ambiguity between the results obtained with the butanolic fraction, the explanation lies in the polarity of the constituent compounds. As they are located within the cell, for an exogenous compound to directly influence mitochondrial activity, there must be an appropriate balance between lipophilicity and hydrophilicity to traverse two cellular barriers: the tumor cell membrane and the mitochondria. Compounds with such physicochemical properties may be present in the crude extract, represented by a wide range of polarities among their phytochemical components; however, they are unlikely to be extracted into the butanolic fraction, which concentrates compounds of higher polarity. Thus, we observed dose-dependent activity of the crude extract in both colorimetric assays, whereas this effect was only observed in the crystal violet assay when using the butanolic fraction. This represents an important selection of cytotoxic compounds in this fraction with apparently low cellular permeation. We can consider the possibility of using this fraction for the treatment of surface tumors (for local action) with fewer systemic effects, thereby providing greater safety.

The wound healing assay allowed us to observe not only the lack of recovery from the injury caused to the cell monolayer but also significant alterations in the morphology of the remaining cells. This clearly demonstrated the cytotoxic activity of both extracts and the extracts in association with the chitosan membrane after 24 and 48 h of incubation.

Apparently, the membrane embedded with 1000 µg/mL of the crude extract was capable of replicating the inhibition of cell viability measured using colorimetric assays, albeit with lower values compared to those obtained by direct inoculation of the extracts into cell cultures. However, the inhibition percentages derived from the butanolic fraction did not differ from those obtained for the control (chitosan membrane free of extracts). This difference is of great importance in the development of a therapeutically useful membrane that can incorporate and release the phytochemical constituents of the active extract. Although chitosan biomembranes are widely used for the conveyance of biologically active substances, their adhesion must be due to their chemical and physicochemical properties (27), which should be strong enough to be incorporated into the membrane, yet weak enough to allow their dissociation and permeation into organic tissues. Thus, it is possible that the highly polar compounds present in the butanolic fraction interacted more intensely with the polar hydroxyl and amine groups in chitosan, hindering the release of these compounds into the cell culture medium.

Thus, despite the significantly subtler effects observed with the crude extract when associated with the membrane, we understand that the membrane was capable of broadly incorporating the constituents of this extract, indicating its potential for therapeutic use. However, future studies should comparatively evaluate the phytochemical composition of the extracts and their derived membranes to further the understanding of the capacity of these membranes to incorporate and release biologically active compounds, especially from the butanolic fraction, which is of special interest.

### Conclusions

Fractionation using n-butanol from a 90% hydroethanolic extract of *A. oleracea* leaves isolated phytochemicals with notable cytotoxic properties and induced morphological alterations in HEp-2 cells. This process revealed distinct cytotoxic mechanisms compared to the full spectrum of compounds in the original extract. These findings suggested further investigative potential for the different fractions obtained through hydroethanolic extraction.

In contrast to the crude 90% hydroethanolic extract, which significantly affected mitochondrial activity in a dose-dependent manner, the butanol fraction showed minimal interference, suggesting superficial or cellular membrane action with low cellular permeation. These cytotoxic and mitochondrial effects were not observed when the cells were integrated into the chitosan membrane.

Moreover, chitosan membranes embedded with 1000 µg/mL of the crude extract were capable of replicating the cytotoxic actions of the pure extract, confirming their potential as a foundation for pharmaceutical formulations to deliver standardized plant extracts for treatment of surface cancers.
